# Novel markers for OLM interneurons in the hippocampus

**DOI:** 10.3389/fncel.2015.00201

**Published:** 2015-06-02

**Authors:** Sanja Mikulovic, C. Ernesto Restrepo, Markus M. Hilscher, Klas Kullander, Richardson N. Leão

**Affiliations:** ^1^Unit of Developmental Genetics, Department of Neuroscience, Uppsala UniversityUppsala, Sweden; ^2^Neurodynamics Lab, Brain Institute, Federal University of Rio Grande do NorteNatal-RN, Brazil

**Keywords:** interneuron, OLM, somatostatin, hippocampus, Chrna2

Oriens-lacunosum moleculare (OLM) cells are a major subclass of hippocampal interneurons involved in controlling synaptic plasticity in Shaffer collateral synapses (Leão et al., 2012) and electrogenesis in pyramidal cell (PC) dendrites (Lovett-Barron et al., 2012). Their firing phase is locked with theta oscillations, which imply a role for these cells in theta rhythmogenesis (Klausberger and Somogyi, 2008; Forro et al., 2015). OLM interneurons also appear to be key in the pathophysiology of epilepsy (Dugladze et al., 2007) and is the most vulnerable interneuron population in models of epilepsy (Dinocourt et al., 2003).

Somatostatin has been frequently used as a molecular marker for identification of OLM cells (Forro et al., 2015). Two recent studies suggest that the OLM cell population is heterogeneous. First, the expression of cholinergic receptor, nicotinic, alpha polypeptide 2 (Chrna2) seems to be restricted to OLM interneurons neurons of CA1 (Leão et al., 2012). Second, a subset of OLM interneurons that expresses the 5HT3a receptor is derived from the caudal ganglionic eminence and do not entrain to gamma oscillations. In contrast, OLM interneurons derived from the medial ganglionic eminence partially phase lock to *in vitro* gamma oscillations and do not express 5HT3a receptors (Chittajallu et al., 2013). Further, other dendritic targeting interneurons in the hippocampus also express somatostatin (Lovett-Barron et al., 2014). Hence, functional studies of OLM cell in hippocampal function have been targeting a relatively heterogenous cell population.

Moreover, one of the most widely used somatostatin-Cre mouse lines, the Som-Ires-cre line (Taniguchi et al., 2011), shows rather unspecific Cre activity in the neocortex, targeting both dendritic and somatic projecting interneurons (Hu et al., 2013). While no study have yet systematically characterized Cre activity in the hippocampus of this somatostatin-Cre mouse line, our own observations indicate a heterogenous activity pattern also in the hippocampus. We crossed somatostatin-Cre males with females of the Ai14 reporter line, to generate double transgenic progeny in which somatostatin positive cells express td-tomato (Figure 1A). Cre-driven td-tomato expression in somatostatin-Cre mice was not restricted to OLM cells. We found several PCs labeled and observed td-tomato+ cell bodies across all CA1 layers as well as cells labeled in the dentate gyrus and CA3. Further, the firing properties of CA1 neurons expressing td-tomato in somatostatin-Cre mice were heterogeneous (Figure 1A). Td-tomato positive recorded cells were classified into regular-(RS), slow-(SS), and fast-spiking (FS) neurons, using clustering method as described previously (Hu et al., 2013). The proportion of RS, SS, and FS in CA1 neurons expressing td-tomato was 63% (20/31), 31% (10/31), and 6% (1/31), respectively.

**Figure 1:**
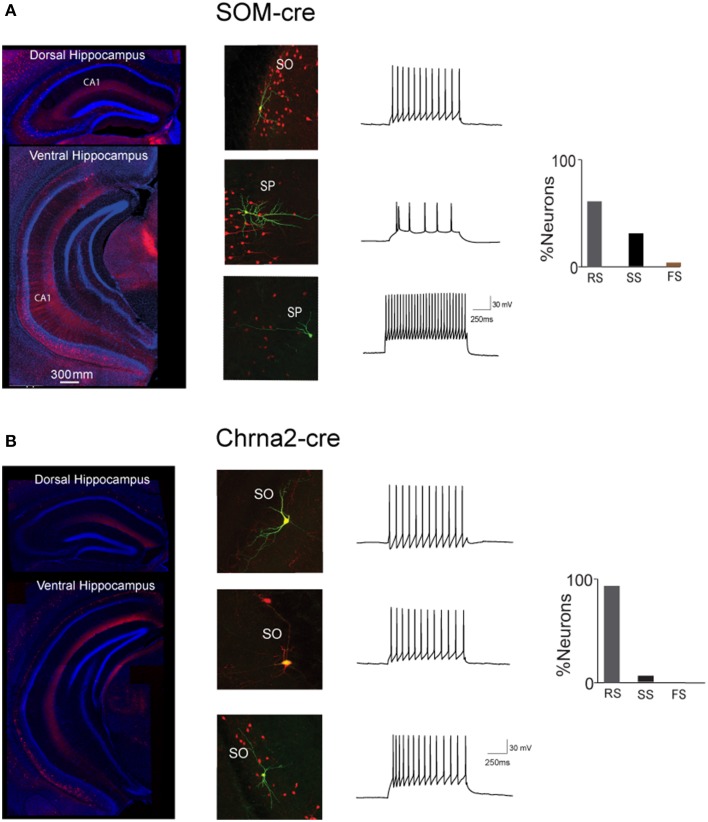
**Differential cell labeling in Somatostatin-cre and Chrna2-cre mouse lines in the CA1 hippocampal region. (A)** (Left) Somatostatin-cre-tomato animal. Tomato expression in the dorsal and ventral parts of the hippocampus. (Right) Examples of neurons recorded and filled in Somatostatin-cre animal. Note cell diversity including regular-(RS), slow-(SS), and fast-spiking neurons and their location in both stratum oriens (SO) and stratum pyramidale (SP). **(B)** (Left) Chrna2-cre-tomato animal. Tomato expression in the dorsal and ventral parts of the hippocampus. Note increasing number of cells along the dorso-ventral axis. (Right) Examples of neurons recorded and filled in Chrna2-cre animal (Leão et al., 2012).

The classification of CA1 interneurons as dendritic and perisomatic targeting may oversimplify the analysis of CA1 networks. PCs possess basal and apical dendrites that are supplied by different inputs (Otmakhova et al., 2002). In addition, single primary dendrites are heterogeneous in their expression of ion channels and synaptic receptors (Magee, 1999; Otmakhova et al., 2002), which drastically alter the local computation of excitatory and inhibitory synaptic inputs (Leão et al., 2012; Lovett-Barron et al., 2012). Hence, different subtypes of somatostatin positive neurons differ considerably in their activity and function (Müller and Remy, 2014). For example, in the CA1, somatostatin positive bistratified cells innervate the proximal apical and basal PC dendrites while OLM cells almost exclusively innervate distal PC dendrites (Leão et al., 2012; Lovett-Barron et al., 2012; Müller and Remy, 2014). These differences in innervation (apart from being pathway specific) associated to a larger density of ion channels that mediate hyperpolarisation-activated currents at distal apical dendrites may indicate that rebound depolarisations could be triggered by OLM but not bistratified cell inhibition (Leão et al., 2011). Rebound depolarisation at the stratum lacunosum-moleculare was indeed observed in our voltage-sensitive dye recordings after OLM cell activation (Leão et al., 2012). Hence, different subtypes of somatostatin positive neurons in the CA1 may act distinctly in controlling input/output functions of PCs. Expression of somatostatin also changes during hippocampal development. While more restricted to the stratum oriens in young/neonate mice, somatostatin is more widespread in adulthood, especially in the ventral hippocampus (http://mouse.brain-map.org/experiment/show/1001). An immunohistochemistry study has also shown that adult animals exhibit larger number of identified somatostatin positive cells in stratum pyramidale (Kosaka et al., 1988). Thus, using somatostatin expression to functionally isolate CA1 interneurons (e.g., in optogenetic experiments) may not be the optimal approach to understand the role of distinct interneurons in CA1 function.

We recently described a transgenic mouse line that expresses Cre predominantly restricted to OLM cells of the CA1 and subiculum (Figure 1B, Leão et al., 2012). In our Tg(Chrna2-cre)1Kldr mice, Cre is expressed under the control of the *chrna2* promoter. Ninety two percentage (155/168) of all recorded/filled cells in the CA1 of these mice could be classified as OLM and little or no Cre activity was observed in the dentate gyrus or CA3 (Leão et al., 2012). *In situ* hybridization experiments, however, indicate that *chrna2* is expressed in both dorsal and ventral hippocampus, especially at the hilus, the stratum oriens of CA1, the subiculum and, to a lesser extent, CA3 (mouse.brain-map.org/experiment/show/75551460). Hence, it seems that the variability in bacterial artificial chromosome (BAC) transgene technology restricted Cre expression to a very specific subpopulation of Chrna2+ cells. Notably, two other GENSAT BAC lines attempting to use the *chrna2* promoter appear to have a less restricted expression of Cre (Chrna2-Cre_OE25 and Chrna2-Cre_OE29, www.gensat.org).

In addition to the restricted expression in the OLM cells of the CA1/subiculum, Cre expression in Tg(Chrna2-cre)1Kldr was observed to be more significant in the intermediate and ventral hippocampus (Figure 1). This finding suggests that Tg(Chrna2-cre)1Kldr mouse could be used to study differences between dorsal and ventral hippocampus function (Forro et al., 2015). In summary, classical interneuron markers like somatostatin (or parvalbumin to some extent) should be used with caution when striving to separate functionally distinct interneuron populations. Alternative genetic markers are now available that may serve as better tools for investigation of interneuron function.

## Conflict of interest statement

The authors declare that the research was conducted in the absence of any commercial or financial relationships that could be construed as a potential conflict of interest.
